# Are Social Networks Watermarking Us or Are We (Unawarely) Watermarking Ourself?

**DOI:** 10.3390/jimaging8050132

**Published:** 2022-05-10

**Authors:** Flavio Bertini, Rajesh Sharma, Danilo Montesi

**Affiliations:** 1 Department of Mathematical, Physical and Computer Sciences, University of Parma, 43100 Parma, Italy; 2 Institute of Computer Science, University of Tartu, 50090 Tartu, Estonia; rajesh.sharma@ut.ee; 3 Department of Computer Science and Engineering, University of Bologna, 40126 Bologna, Italy; danilo.montesi@unibo.it

**Keywords:** authorship attribution and verification, watermarking, fake profiles, Social Networks

## Abstract

In the last decade, Social Networks (SNs) have deeply changed many aspects of society, and one of the most widespread behaviours is the sharing of pictures. However, malicious users often exploit shared pictures to create fake profiles, leading to the growth of cybercrime. Thus, keeping in mind this scenario, authorship attribution and verification through image watermarking techniques are becoming more and more important. In this paper, we firstly investigate how thirteen of the most popular SNs treat uploaded pictures in order to identify a possible implementation of image watermarking techniques by respective SNs. Second, we test the robustness of several image watermarking algorithms on these thirteen SNs. Finally, we verify whether a method based on the Photo-Response Non-Uniformity (PRNU) technique, which is usually used in digital forensic or image forgery detection activities, can be successfully used as a watermarking approach for authorship attribution and verification of pictures on SNs. The proposed method is sufficiently robust, in spite of the fact that pictures are often downgraded during the process of uploading to the SNs. Moreover, in comparison to conventional watermarking methods the proposed method can successfully pass through different SNs, solving related problems such as profile linking and fake profile detection. The results of our analysis on a real dataset of 8400 pictures show that the proposed method is more effective than other watermarking techniques and can help to address serious questions about privacy and security on SNs. Moreover, the proposed method paves the way for the definition of multi-factor online authentication mechanisms based on robust digital features.

## 1. Introduction

In recent years, various Social Networks (SNs) have been introduced in order to cater to the different needs of users: social interactions (Facebook), professional interconnections (LinkedIn), photo sharing (Instagram), and instant messaging (Whatsapp), to name a few. An important reason for the huge popularity of social platforms among users is the increase in the use of smartphones, which in turn has introduced changes in user habits with respect to multimedia content on SNs [1]. In particular, many social platforms are predominantly used through mobile devices, for example as Instagram and WhatsApp.

On the flip side, illicit actions across these SNs are constantly growing, such as illegal copying, identity impersonation, and pedopornography [2]. In particular, the creation of fake profiles is an important problem across these SNs, and has seen a sharp increase in recent times [3]. In fake profiles, known as *impersonating profiles*, a malicious user copies images from another profile and claims to be the person in the profile’s pictures. A natural solution to hinder the process of fake profile creation is through encouraging authorship attribution and verification through images. In particular, firmly tying an image to its creator or being able to prove ownership of a particular image is crucial to uncovering illegal activities such as those listed above.

Broadly, two techniques, namely, watermarking and steganography (both techniques belonging to *information hiding*), are used to embed messages in digital content [4]. Watermarking is the practice of imperceptibly altering digital content to embed a mark. Watermarking is the most commonly used technique for owner identification, proof of ownership, authorship attribution, and verification [4], and is generally used in the domain of digital copyright protection [5]. Steganography is the practice of undetectably altering digital content to embed a secret message. As steganography techniques can be used with a mark and not only with a secret message, we refer to watermarking approaches both for conventional watermarking and steganography throughout this work. In particular, we investigated the possibility of using watermarking in the context of online social platforms to tackle the problems of authorship attribution and verification.

### 1.1. Problem Statement

In this study, the scope of digital content is limited to images, as image sharing is the most commonly observed behaviour on SNs. In particular, this work explores the possibility of watermarking the images being uploaded on SNs, and answers the following three questions:

1. **Social Network Watermarking—***Are SNs watermarking our images?*
  In Section 5, we present the results of our experiments to understand whether SNs mark the images being uploaded on their social platforms. The relevance assumed by SN platforms in multiple aspects of daily life makes this a topic of extreme importance; however, it is poorly addressed in the literature. Our findings revealed that, in general, SNs do not perform any watermarking techniques on images. Out of the thirteen SNs analysed, we found that only Facebook changes the metadata associated with images to any extent. We performed extensive tests in order to verify whether these changes can be imputed to a watermarking function.
2. **User-Explicit Watermarking—***Can conventional watermarking techniques pass through SNs unaffected?*
  In Section 6, we explore various watermarking algorithms as a tool for reliably marking images to be uploaded on SNs. Existing studies cover a limited number of SN platforms and use low-resolution images, making their assessments incomplete and not reflective of real scenarios where a single user has more than one account on multiple SN platforms. Through our analysis, we found that there is no single watermarking technique that can be successfully used across all of the selected SNs.
3. **User-Unaware Watermarking—***Are we unknowingly watermarking our images?*
  Taking inspiration from previous works where researchers exploited sensor imperfections to extract the fingerprint to identify a smartphone [6,7,8], we analysed whether the cameras of smartphones through which images are taken can be used to create a watermark. According to conventional watermarking literature [4], the proposed method is *invisible* and *detectable* and belongs to the *fragile* and *blind* categories.
  In practice, the fingerprint of the smartphone camera can be extracted from the images without altering the device. This represents the main contribution of our work, and paves the way for addressing several different crucial subtasks related to SNs such as user profile resolution and multi-factor online authentication. In Section 3, we provide more details about our method. We demonstrate in Section 7 that the proposed method is robust enough, despite the uploading and downloading processes of SNs downgrading the shared images.

### 1.2. Contributions

The crux of this paper’s central idea is that embedding a watermark into shared pictures helps users prove their ownership, subsequently avoiding privacy violations even when the information held by SNs on the fake profiles that re-shared those pictures is not reliable. To the best of our knowledge, this is the first study to investigate the use of the PRNU-based method as a reliable image watermarking technique on these thirteen SNs. First, the results of our analysis showed that no profile-dependent watermark operations are performed by the SNs. Second, the results showed that the main problem with conventional image watermarking algorithms is that they fail to pass through all of the thirteen SNs considered in this study. These two preliminary results support the proposed method for authorship attribution and verification based on a well-known technique that is commonly used in digital forensic or image forgery detection activities.

**Figure 1:**
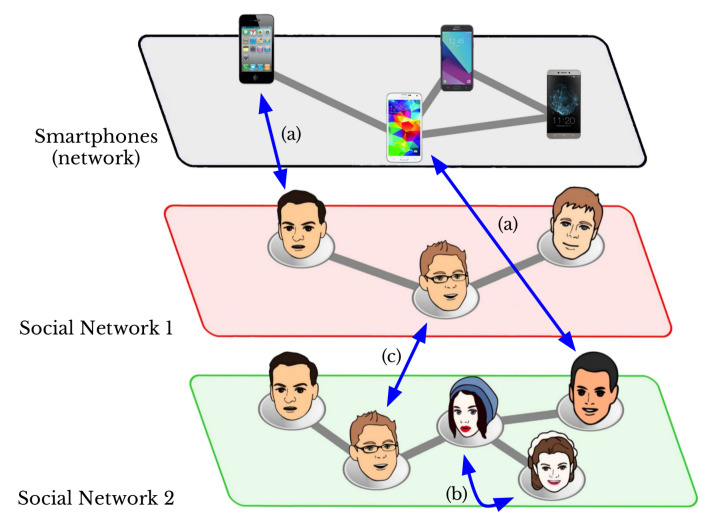
User-unaware watermarking’s three main tasks: *profile attribution* (**a**), *intra-layer user profile linking* (**b**), and *inter-layer user profile linking* (**c**).

In particular, the most important finding of this study is that even if the author of an image does not consciously perform any *user explicit watermarking* technique, the smartphone camera embeds its characteristic fingerprint in every taken picture. Likewise, as in a conventional watermarking approach, the method allows the rightful owner of the images to be identified, and it can be successfully applied across all thirteen investigated SNs. Figure 1 shows users’ smartphones and two different SNs where users shared images taken from their smartphones. We used the fingerprints left by the smartphone’s camera to deal with the following problems:

1. *Profile Attribution—*the task of matching a user profile to the right smartphone within a set of devices through a set of shared images, i.e., case (a) in Figure 1.
2. *Intra-layer User Profile Linking—*the task of deciding whether a restricted set of user profiles within the same SN belongs to the same user, i.e., case (b) in Figure 1.
3. *Inter-layer User Profile Linking—*as in the previous task, this task attempts to match user profiles that belong to different SNs, i.e., case (c) in Figure 1.
4. *Fake Profile Detection—*the task of identifying unauthorized clones of user profiles. This task is a corollary of all the previous tasks, as an untrusted/fake profile can be linked to a verified one using the shared images.

It is noteworthy that the two types of *user profile linking* tasks described above are not always possible to achieve using conventional watermarking techniques, as in certain cases it is necessary to have the original image in order to extract the watermark. The proposed method is robust enough in spite of the fact that the images are downgraded during the process of uploading and downloading them on the SNs. Moreover, it is possible to address authorship attribution and verification on SNs without having the original images or altering the device.

Using the PRNU-based method, which is one of the most robust methods for device identification [9], in this paper we show that it can be used to solve cross-platform authorship attribution and verification, which is not possible with conventional watermarking algorithms. In particular, the proposed approach shows that, in addition to the classical digital forensics context, the method can be used to tackle privacy and security issues on online SN platforms. Moreover, the proposed method paves the way for the definition of multi-factor online authentication mechanisms based on robust digital features.

The remainder of the paper is organized as follows. Section 2 presents the literature on three different domains, image watermarking, smartphone fingerprinting, and user profile linking, in the context of SNs. The proposed approach is presented in Section 3, whereas the experimental settings (i.e., the selected Social Networks and the characteristics of the used images) are briefly described in Section 4. The investigation results regarding possible built-in watermarking techniques adopted by SNs are presented in Section 5. In Section 6, the outcomes of the *user-explicit watermarking* techniques on SNs are discussed, while the results using the proposed PRNU-based method for *user-unaware watermarking* are presented in Section 7. A discussion and concluding remarks are provided in Section 8 and Section 9, respectively.

## 2. Related Works

In this section, we describe the literature from three domains, all of which are relevant for full comprehension of our work. First, we focus on conventional image watermarking techniques on SNs. Next, we present various approaches for uniquely identifying smartphones. Finally, we explain methods for associating user profiles in SNs, which is the main outcome of this work.

### 2.1. Image Watermarking in Social Networks

Image watermarking techniques embed a mark in a visually imperceptible way for authentication and copyright protection tasks [10]. According to the embedding domain [11], these techniques can be classified as *spatial* and *transform domain* watermarks.

The methods in the *spatial domain watermarking* class directly modify the image pixels by acting on the bit value. The simplest approach embeds the watermark in the Least Significant Bits (LSB) [12]. The Intermediate Significant Bit (ISB) method [13] improves the LSB method and defines the watermarked location according to the range of each bit-plane. The patchwork method [14] is another spatial domain approach where the watermark is embedded into the image by changing the pixels’ brightness. In general, the simple implementation of these spatial domain methods implies lower robustness against affine transformations and image processing attacks [15].

The methods in the *transform domain watermarking* class apply a transformation to the original image and exploit the transformed coefficients to embed the watermark. There are four main techniques in transform domain: Discrete Cosine Transform (DCT), Discrete Wavelet Transform (DWT), Discrete Fourier Transform (DFT), and Singular Value Decomposition (SVD). Typically, DCT algorithms segment the image into blocks and modify a set of selected coefficients [16]. Similarly, in DWT techniques the original image is decomposed into three spatial directions (i.e., horizontal, vertical, and diagonal), then the watermark is embedded in the wavelet coefficients [17]. DWT algorithms are computationally efficient and the visual artefacts introduced are less evident compared to DCT. DFT algorithms employ the Fourier transform, which offers robustness against geometric attacks [18]. DFT decomposes the original image in phase and magnitude representation. Then, the mark is embedded into the magnitude representation. SVD is one of the most powerful numerical analysis techniques [19]. In particular, SVD allows the mark to be embedded into the singular matrix of the frequency domain or spatial domain coefficients with much less loss of information [20]. Moreover, in order to exploit its various properties SVD can be combined with the other techniques: DWT-SDV [21], DFT-SVD [22], and DWT-DFT-SVD [23]. As the transform domain methods lead to robust watermarking [24], in our tests we used methods that belong to the *transform domain watermarking* class.

Recently, researchers have investigated various watermarking techniques for use on SNs as well as potential attacks and corresponding solutions [25]. In [26], the researchers proposed a dual watermarking scheme for Facebook and Google+ by partially redesigning the SN uploading service, which may not be feasible. In [27], the authors considered Facebook as a closed system and tested different steganography methods. A watermarking method based on wavelet decomposition proposed in [28] was successfully tested by simulating different attacks that can be performed over SNs. In [29], the authors proposed a method based on DWT coefficients; however, the tests were performed on images with low resolution that were not compressed during the uploading process on the selected four SNs. Similarly, in [30], a method based on DCT transformation was proposed for Facebook using images that the SN did not resize. A method based on a backpropagation neural network was proposed in [31]; however, the authors did not perform any test on real SNs and used images with a very low resolution in comparison to the resolutions of current smartphone cameras. An orthogonal approach was presented in [32], where the author proposed using SN the hashtag symbol (“#”) to hide information through the images uploaded on Twitter and Instagram. To the best of our knowledge, this is the first work where images with high resolution were used to investigate image watermarking techniques across all thirteen major SNs.

### 2.2. Fingerprinting the Smartphone Devices

Smartphones are becoming more and more pervasive in daily activities. Recently, researchers have proposed methods for identifying and fingerprinting smartphones by exploiting personalized configurations [33], touchscreen interaction [34], and the on-board camera [35]. Today, smartphones are equipped with a number of sensors. These sensors are produced according to industrial standards, which ideally makes them identical; however, each sensor has an imperfection that makes it unique and identifiable [36]. Microphones and speakers, through playback and recording of audio samples, were exploited in [8]. In [6], the authors proposed a technique using the integrated accelerometers in smartphones to identify individual mobile phones. An improvement compared to [6] was proposed in [7], where the authors used the speakerphone–microphone along with the accelerometer.

Among all the sensors described above, the most widely investigated sensor in the field of digital forensic is the camera [37]. The reason for this is that the camera contains several components inside it which can be used to identify the source camera. The chromatic aberration introduced by the lens was exploited in [38], whereas in [39] camera identification was achieved through the colour filter array (CFA), which was used to recognize a counterfeit image in [40] as well. In [41], the authors proposed a sensor-based method exploiting Photo-Response Non-Uniformity (PRNU) to successfully distinguish cameras of the same model. A PRNU-based method able to operate with different image sizes was presented in [42]. In [43], Castiglione et al. classified all the various changes made by the SNs to uploaded images, which commonly causes a loss of effectiveness of these camera fingerprinting methods. Later, in [44], the same authors demonstrated the robustness of their PRNU-based method on images downloaded from six SNs and from several online photo-sharing platforms.

Recently, the PRNU-based method has been used to perform blind clustering on both pristine images and compressed images downloaded from SNs, addressing the source camera identification task [45,46]. In [47], the authors investigated the possibility of privacy leaks related to Photo-Response Non-Uniformity noise. In particular, they claimed that by using the camera fingerprint a malicious user could identify the owner of a device by matching the fingerprint with the noise in images crawled from an SN account, further emphasising the effectiveness of the PRNU-based method. In [48], the authors proposed a PRNU-based approach to tackle morphing attacks, which pose a severe security risk to facial recognition systems. The PRNU-based method has been studied in an image forgery context as well. In particular, in [49] the authors carried out both analytical and empirical studies on the impact of different camera sensitivity settings on PRNU-based digital forensics, proposing an ISO-specific correlation prediction process for forgery detection. In [50], the authors explored the effectiveness of convolutional neural networks in predicting PRNU correlations under complex backgrounds in order to improve the accuracy of forgery localisation. As the effectiveness of the PRNU-based approach has been widely demonstrated in several contexts [51], including on large scale image datasets [52] and multimedia forensics [53], we decided to use it for source camera fingerprinting on SNs.

### 2.3. User Profile Linking in Social Networks

Trust is a fundamental ingredient in SNs [54], and a significant increase in the number of impersonating fake profiles has resulted in various solutions for deciding whether a given user profile is an unauthorized clone of another [55]. In [56], the authors proposed a three layered tool for Facebook which was able to (*i*) identify suspicious users, (*ii*) expand basic privacy settings, and (*iii*) warn the user against malicious applications. Similarly, a graph-based framework for detecting fake profile attacks was proposed in [57].

The more generic user profile linking task allows matching different user profiles belonging to the same user, which is analogous to the missing data problem in multilayer networks [58]. An invasive and device-dependent solution exploits the log information stored on the device’s internal memory during the use of the SN application [59]. The more effective solutions for user profile linking exploit the information and multimedia content that transits SNs. A framework for user profile linking based on the profile’s attributes was proposed in [60], while in [61] the authors combined tags and user ID to match users’ profiles across different social tagging systems. The solutions proposed in [62] and [63] match user profiles by using information about users’ identities without compromising their privacy. In [64], the authors presented a method that combines profile attributes and social linkage to outperform common attribute-based approaches, whereas in [65], network attributes and profile attributes were combined to improve a traditional identity search algorithm. A weighted ontology-based user profile linking technique was proposed in [66]. In order to improve the performance of the attribute-based approach proposed in [67] and [68], the authors proposed machine learning techniques to match user profiles across multiple SNs. Typically, these approaches fail if a malicious user falsifies the information stored in the fake profile, as usually happens. On the other hand, image-based approaches have recently provided promising results both without prior knowledge of the source camera [69] and in presence of outliers [46].

## 3. Proposed Approach

In this section, we introduce the proposed approach used to answer the three research questions presented in Section 1.1. In particular, we provide details about the experimental protocol followed to understand whether SNs mark the images being uploaded on their platforms, we describe the characteristics of the algorithms used to determine whether conventional image watermarking techniques can reliably be used on SNs for marking the images to be uploaded, and finally we describe the PRNU-based method used to verify whether the noise left by the sensor of the smartphone’s camera can be successfully used to watermark images uploaded on SNs. Details about the experimental setting (i.e., the selected SNs and the characteristics of the used images) are provided in Section 4.

The first direction of investigation concerns whether SNs are watermarking our images. The context of watermarking can be broadened in terms of changes performed by a particular SN, such as a different name and metadata associated with the image after being uploaded on the SN. Thus, different comparisons can be performed among uploaded and downloaded images. First, we performed preliminary analyses of the compression of the images in term of the applied compression. Next, we compared the names, contents (i.e., the image without metadata), and metadata of the uploaded and downloaded images on each SN. Finally, we executed additional analysis on Facebook metadata, including the Content Delivery Network (CDN), an intermediate layer of proxy servers distributed globally. Typically, CDNs are widely used by high traffic websites, as they allow them to provide digital content with high availability and performance. The obtained results on *social network watermarking* are discussed in Section 5.

There are a number of conventional watermarking tools, however, to answer the question *Can conventional watermarking techniques pass through SNs unaffected?* therefore, we selected open-source or freeware tools that work with the image file formats accepted by SNs (i.e., jpg and png). These tools can be categorized into *spatial* and *transform domain watermarking* algorithms. The first category includes easy to implement and low complexity methods. On the other hand, these watermarking tools present weaknesses including weak robustness and low security. The methods in the *transform domain watermarking* class are widely applied and can reach a good balance between robustness and imperceptibility. All of the selected algorithms belong to the *invisible* watermarking class, which means that the embedded watermark is not noticeable by the user. The selected tools and the obtained results are presented in Section 6.

In both *social network watermarking* and *user-explicit watermarking*, we created two different user profiles for each of the SNs (i.e., *P*${}_{1}$ and *P*${}_{2}$). This had two advantages; first, it allowed us to compare the original image with the downloaded one, that is, the “Original vs. Shared” case, and second, we were able to compare the same image after being uploaded twice on two different user profiles, that is, the “Shared *P*${}_{1}$ vs. Shared *P*${}_{2}$” case.

The third contribution of our work investigated the possibility of exploiting sensor imperfections in the camera through which the images had been taken to create a watermark to be used on SN platforms, that is, *user-unaware watermarking*. The sensor pattern noise (SPN) due to manufacturing imperfections is considered a unique characteristic that can fingerprint a source camera, and has been shown to outperform other techniques [9]. Our contribution in this paper is to show how it can be used for images shared online.

**Figure 2:**
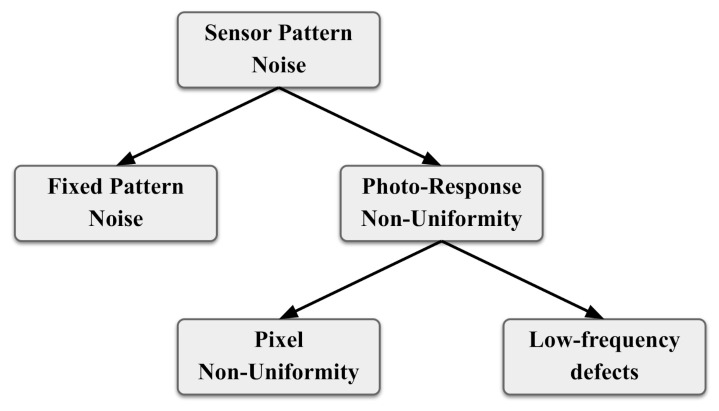
Sensor patter noise classification.

The SPN contains Fixed Pattern Noise (FPN) and Photo-Response Non-Uniformity (PRNU) noise (Figure 2). The first is created by dark currents when the sensor array is not exposed to light and is affected by ambient temperature and exposure. Moreover, as the FPN is an additive component, cameras suppress it automatically. The PRNU represents the main component of the SPN, and is generated primarily by Pixel Non-Uniformity (PNU), that is, the different sensitivity of pixels to light, which is caused by the inhomogenity of silicon wafers and imperfections. The PNU is regular and systematic, and it is unlikely that sensors from the same wafer will present correlated PNU patterns. The second negligible component of the PRNU is low-frequency defects due to light refraction on dust particles and optical surfaces and zoom settings, and is not a characteristic of the sensor. Camera fingerprinting using PRNU-based methods has been proven to provide very reliable results [9]; however, to the best of our knowledge there is a shortage of studies investigating the possibility, in addition to tackling forensic issues, of addressing security and privacy-related problems such as authorship attribution and verification of shared images as well.

We exploited a PRNU-based method [41] to extract the dominant part of the regular noise, which we define as the *user-unaware watermark*. In particular, we tested several different algorithms, including the Diffusion Anisotropic [70], Diffusion Isotropic [71], Block Matching 3D (BM3D) [72], Wavelet Soft Threshold [73], Wavelet Hard Threshold [74], and Wavelet Multi Frame [75] algorithms. The BM3D approach provided the best results, especially when combined with the Y channel of the images, which is the luminance component in the YCbCr colour space format. The fingerprint, $FP_{i}$, of a smartphone camera *i* was approximated as the average of the residual PRNUs of *n* pictures captured by that device:

(1) $$FP_{i}=\frac{1}{n}\sum_{j=1}^{n}BM3D\left(Y\left(I_{j}\right)\right)$$

where the function Y() extracts the *Y* channel, BM3D() extracts the PRNU, and the resulting $FP_{i}$ is a matrix with the same size as the original image. In order to evaluate whether a generic image $I_{k}$ had been taken by the same camera, the standard normalized correlation was applied between the PRNU ($N_{k}$) extracted from $I_{k}$ and the fingerprint $FP_{i}$

(2) $$corr\left(N_{k},FP_{i}\right)=\frac{\left(N_{k}-\bar{N_{k}}\right)\left(FP_{i}-\bar{FP_{i}}\right)}{∥\left(N_{k}-\bar{N_{k}}\right)∥∥\left(FP_{i}-\bar{FP_{i}}\right)∥}$$

where $N_{k}$ is equal to $BM3D(Y\left(I_{k}\right))$ and $\bar{N_{k}}$ and $\bar{FP_{i}}$ are scalars that represent the mean value of the $N_{k}$ and $FP_{i}$ matrices. The correlation value can vary from 0 (different source) to 1 (same source). In order to correlate images with different sizes, large images were scaled to smaller ones [46]. The obtained results for *user-unaware watermarking* are discussed in Section 7.

## 4. Experimental Settings

In this section, we describe the Social Networks and the characteristics of the images that were used in our analysis.

The thirteen most popular SNs, as shown in Table 1, were used for our investigation. The selection of these SNs was based on two different criteria, namely, the number of user accounts and the different features offered by the social platforms. All of the selected SNs in total count more than 100 million users, and cover different needs such as social interaction (Facebook), photo sharing (Instagram), blogging (Tumblr), instant messaging (WhatsApp), professional interconnections (LinkedIn and Google Currents), to name a few. Moreover, we decided to include SNs developed outside of the United States and European Union, such as Telegram, VK (originally VKontakte), and WeChat. As we were interested in image watermarking algorithms, in Table 1 we specify the default pixel resolution accepted by each social platform.

All of the SNs accept Joint Photographic Experts Group (JPEG or JPG) images with standard pixel resolution, that is, they have default image sizes (see Column III in Table 1) beyond which the image is automatically scaled to the default resolution. For this reason, we carried out tests for *social network watermarking* and *user explicit watermarking* with three different resolutions: standard, matching the image size of the SN; larger than the standard (4128 × 2322); and smaller than the standard (640 × 480). We used ten different images for each resolution class (standard, large, and small). To evaluate the proposed approach for *user-unaware watermarking*, we selected six smartphones from three different brands with three pairs of identical models (Table 2). Moreover, a different user profile was created on each SN. The reason for picking identical models was to verify whether the test could work across two different phones of the same model, as identical models mount identical components. For each device we considered both the front and rear camera, as these usually present different characteristics in term of sensors, quality, and resolution. The *iPhone 6* and *iPhone 6 Plus* both have an identical front and rear camera. For each camera of each device, we took 700 photos. In total, the dataset consisted of 8400 images (and is available from http://smartdata.cs.unibo.it/datasets#images, accessed on April 10 2022 ). Then, for each camera we kept a subset of 50 original images and uploaded and downloaded 50 images on each of the thirteen SNs shown in Table 1.

## 5. Social Network Watermarking

In the following subsections, we describe all the tests carried out with respect to *social network watermarking*.

### 5.1. Preliminary Analysis

We observed that when an image is uploaded to an SN, it is compressed by the JPG compression algorithm adopted by the platform. The compression is due to the optimization of the image by the SN. In particular, the quantization matrix coefficients in the JPG compression algorithm control the compression ratio. This means that any uploaded images may have different file sizes after downloading compared to the original. To the best of our knowledge, SNs do not publish any information about the image processing algorithms being used inside their platforms; thus, they can be conidered a “black box”. For this reason, we investigated the behaviour of the selected SNs using images from three different resolution classes, namely, standard (see Column III in Table 1), large (4128 × 2322), and small (640 × 480).

Our findings pointed out that for each resolution class, Flickr and Google Currents do not apply any compression and preserve the original image size. On average, VK applies a very low compression to standard images, and the ten remaining SNs compress standard and small images by a percentage ranging from 30% to 76%. Generally, large resolution images are strongly compressed by all SNs, except for Flickr and Google Currents, by more than 80%.

### 5.2. Image Comparison

In this section, we present the results of four different kinds of comparisons of images before and after uploading to SNs in order to investigate whether the SNs perform any watermarking activity on the uploaded (shared) images.

- *Name comparison*—This test allowed us to understand whether SNs change the names of uploaded images.
- *Full comparison*—A full comparison was performed by exploiting the *Secure Hash Algorithm* version 1 (SHA-1) to find differences between pairs of images. SHA-1 uses an image as input and produces a unique 160-bit message digest. Any change in the content or metadata of the image implies a different digest in the output.
- *Content comparison*—This test was performed by using a bit by bit comparison of the image’s content, excluding the metadata. The test compared two images in binary representation to highlight the differences between pixels.
- *Metadata comparison*—Metadata are data providing extra information about one or more aspects of a file. Images’ metadata is specified by the *Exchangeable image file format* (Exif) standard, which includes information such as time, location, camera settings, descriptions, and copyright information.

First, the 30 images for each resolution class described in Section 4 were uploaded and downloaded on both *P*${}_{1}$ and *P*${}_{2}$ profiles on each SN. Then, for each SN we performed two different kinds of test. In the first, we compared the original images with the downloaded ones, i.e., the “Original vs. Shared” case. In the second, we compared the shared images on different profiles, i.e., the “Shared *P*${}_{1}$ vs. Shared *P*${}_{2}$” case. For each SN, we wanted to examine whether the changes reverberated in the same way across different profiles or whether they were unique for each profile.

**Name Comparison**—We investigated how the SNs change image names and whether the same image receives different names if shared on different user profiles. Among all of the SNs, we found that Google Currents does not change the original name even when the same image is shared on two different profiles. All other SNs use a specific encoding for image names; however, no significant changes were observed. The Pinterest name format is the most interesting; the original name is not preserved, and the image receives the same name when shared on different profiles irrespective of the resolution class. This means that while the name could be a good candidate for watermarking images, it is quite short and the watermark could be easily removed or changed.

**Full Comparison**—For this comparison, we used the SHA-1 algorithm to check whether the integrity of the original image is preserved. If SHA-1 produces different digests, this means that the input image underwent changes during the uploading and downloading process. The “Original vs. Shared” comparison is consistent with the previous results. In particular, the compression applied by the other SNs changes the images; thus, the produced digests are different among original and shared images except for the two SNs that do not apply any compression, namely, Flickr and Google Currents. The “Shared *P*${}_{1}$ vs. Shared *P*${}_{2}$” comparison produces the same digest, except for Facebook and LinkedIn. These two SNs return different digests for all resolution classes when images are shared on different profiles. Through the following two comparisons we more carefully investigated whether Facebook’s and LinkedIn’s unusual results are due to changes to the content or to the metadata.

**Content Comparison**—In this test, we compared images through a bit by bit difference operation. If two images have the same content, the difference produces a full-zero matrix. *Content comparison* narrows the field to the pixels’ value, excluding the metadata. The “Original vs. Shared” results are consistent with the *full comparison* results. If the SN does not apply any compression, such as Flickr and Google Currents, the content of the original image matches bit by bit with the content of the shared one, while the compression used by the other SNs has obvious effects on the bit-level. The “Shared *P*${}_{1}$ vs. Shared *P*${}_{2}$” case, provides our first important result. Because the image shared on *P*${}_{1}$ matches bit by bit with the same image shared on *P*${}_{2}$ for all SNs, we can infer that all of the selected SNs do not apply any watermarks to the content of images [10].

**Metadata Comparison**—Metadata summarize basic information about the associated file; the *exif* standard defines the metadata attributes of digital images The full attributes list can be categorized by the date and time information, static and dynamic camera settings, general descriptions, copyright information, and thumbnail (a smaller version of the image for indexing and previewing) [76]. Our analysis of the full attribute list for each SN gave rise to several interesting results. Flickr and Google Currents preserve all of the original attributes, whereas, Twitter erases all of them. Tumblr removes only the thumbnail-related fields, while all remaining SNs preserve only a few subsets of the original attribute list, mainly concerning general descriptions and static camera settings. In most of the cases, this subset does not include any GPS information.

We performed further investigation of LinkedIn and Facebook, as these are the only case in which the metadata changes when the same image is shared through different user profiles. LinkedIn uses a subset of the full attribute list and changes certain non-significant attributes, which cannot be related to a watermark as they do not produce a unique identifier for those attributes, only a standard string. On the other hand, Facebook substitutes a set of attributes using the *Information Interchange Model* (IIM), a set of metadata attributes defined by the International Press Telecommunications Council (IPTC). Three of the new attributes receive an alphanumeric character value. In particular, the “Special Instructions” attribute does not change if the same image is shared on two different profiles, as the other two (i.e., “Current IPTC Digest” and “Original Transmission Reference”) are strictly related to the user profile that shares the image. However, we cannot claim that these are used for watermarking purposes. For this reason, we further investigated the Facebook metadata case by performing additional tests. In particular, we studied the following three crucial aspects:

- *Time test*—Images were uploaded and downloaded twice on the same profile after allowing certain period of time to elapse, with the aim of determining whether the added metadata were time-dependent.
- *Sharing test*—Images were uploaded on profile *P*${}_{1}$ and downloaded three times: from *P*${}_{1}$, from *P*${}_{2}$ (which had visited *P*${}_{1}$), and from *P*${}_{2}$ (which had shared the images on the ”wall”). The aim of this test was to determine whether the added metadata were sharing-dependent.
- *Location test*—CDN provides digital content from locations closer to the user. As it was unknown which CDN node served a particular request, we used a VPN to repeat the *sharing test* while forcing one of the two profiles to be located in the following countries: Russia, China, the United States, and the United Kingdom. The aim was to determine whether the added metadata were location-dependent.

In all three new tests, the previous *name comparison*, *full comparison*, and *content comparison* produced the same results. The *metadata comparison* outcome was much more interesting. In the *time test*, the “Special Instructions” attribute was the same even after 24 h, while “Current IPTC Digest” and “Original Transmission Reference” received different values after only a few seconds. In the *sharing test* and *location test*, all three attributes preserved the same values. This suggests that while the added metadata are both profile-dependent and time-dependent, they are neither sharing-dependent nor location-dependent.

As of the time of writing, the *social network watermarking* test indicates that none of the selected SNs apply any profile-dependent watermarks visible outside the network. Facebook introduces certain suspicious values in three new metadata attributes. However, these metadata can be easily erased or modified, and cannot be considered a good solution for authorship attribution and verification purposes.

## 6. User-Explicit Watermarking

In this section, we present the results of several conventional image watermarking techniques on the SNs considered in this study. The goal was to determine whether these approaches can reliably be used on SNs for marking images to be uploaded. We performed our analysis on the set of SNs and the images described in Section 4. For our tests, we selected the following thirteen algorithms: (A1) *BlindHide* [77], (A2) *HideSeek* [77], (A3) *FilterFirst* [77], (A4) *BattleSteg* [77], (A5) *Dynamic FilterFirst* [77], (A6) *Dynamic BattleSteg* [77], (A7) *F5* [78], (A8) *OpenPuff* [79], (A9) *OpenStego* [80], (A10) *Secretbook* [81], (A11) *SilentEye* [82], (A12) *SteganPEG* [83], and (A13) *Steghide* [84].

**Figure 3:**
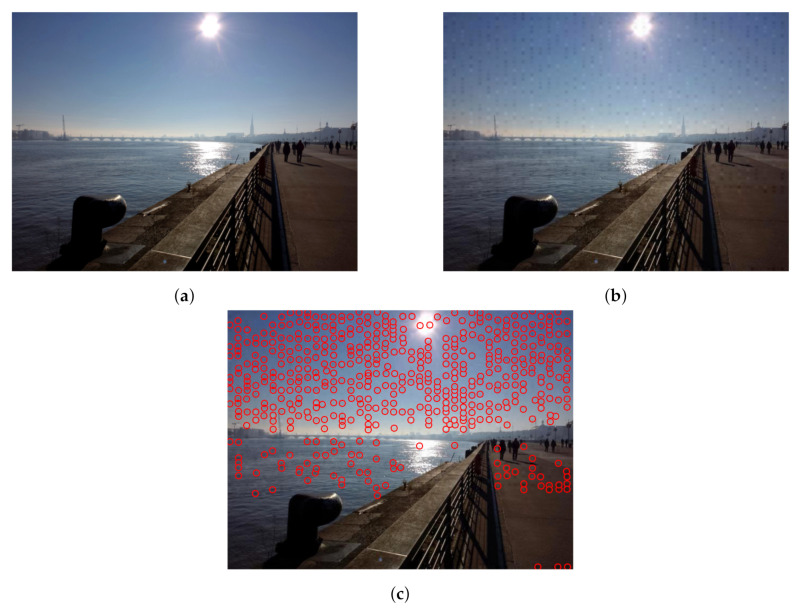
The original image (**a**), the SilentEye outcome (**b**), and the same SilentEye outcome with red circles highlighting artefacts, particularly evident in the sky (**c**).

Table 3 shows the results of the watermark extraction process for each resolution class. In particular, we denote with a • symbol those extraction processes which successfully retrieved the original watermark from the downloaded images. SilentEye and Steghide were not able to embed the watermark in large images, and SteganPEG returned an “image capacity exceeded” error with small images. For these reasons, we considered these three algorithms to have failure. As Flickr and Google Currents do not apply any compression, the watermark was preserved for each watermarking algorithm and resolution class. None of the selected methods was able to create a robust watermark able to successfully pass through Instagram and LinkedIn. Experiments further revealed that spatial domain-based watermarking methods such as BlindHide, HideSeek, FilterFirst, BattleSteg, Dynamic FilterFirst, Dynamic BattleSteg, and SteganPEG are too weak to be applied to shared images on SNs. The other methods, such as F5, OpenPuff, SecretBook, and SilentEye, obtained better results with standard and small images. However, SilentEye heavily transforms the image, which appears visually modified and degraded to the user. Figure 3 shows the effects of the SilentEye algorithm; the watermarked image (Figure 3b) is affected by diffuse noise and artefacts compared with the original one (Figure 3a). The vertical artefacts are highlighted with red circles in Figure 3c, particularly evident in the sky region of the picture where the colour is more bright and uniform.

Based on our findings in the *user-explicit watermarking* test, *transform domain watermarking* methods represent a good candidate for applying personal watermarks to images shared on SNs. However, as shown in Table 3, there is no single watermarking technique that can be successfully used across all SNs. Moreover, the user has to act consciously to embed a watermark into his/her personal photos, which is less likely to happen in scenarios involving instant picture sharing through mobile social platforms such as Instagram and WhatsApp.

## 7. User Unaware Watermarking

Smartphones have contributed significantly to increasing the number of images being shared on SNs [1]. For this reason, in this section we explore whether noise left by the sensors of smartphone cameras can be successfully used to watermark images being uploaded on SNs. In this section we discuss the results of three different tests, namely, *profile attribution*, *intra-layer user profile linking*, and *inter-layer user profile linking* (see Section 1.2) carried out on the downloaded images.

**Figure 4:**
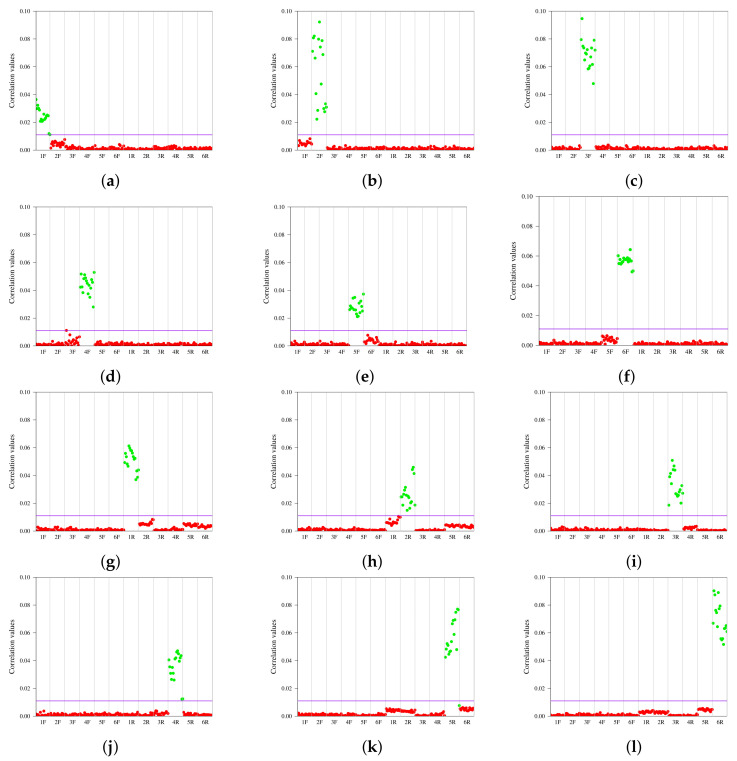
Correlation results for each of the twelve fingerprints: six for the front cameras (**a**–**f**) and six for the rear cameras (**g**–**l**); *X*F and *X*R identify the front and the rear camera, respectively, of a smartphone *X*.

First, in order to provide a benchmark of the effectiveness of *user-unaware watermarking*, we tested the method presented in Section 3 using the original images. The results are shown in Figure 4. The graphs in the first two rows represent the front cameras of each device, while those in the second two rows represent the rear cameras. For each smartphone camera in Table 2, we used a **training set** of 33 images (two-thirds) to define FP and a **test set** of 17 images (one-third) to compose the set of images to be correlated. In accordance with [85], a training set of 20 is sufficient to obtain good results. Each graph represents the values obtained by correlating a specific $FP_{i}$ with all of the images in the twelve test sets. High values were achieved when the $FP_{i}$ and the test set were from the same source (green dots). All obtained correlation values were in the range from 0 to 0.1. However, a heuristic threshold of 0.011 (purple horizontal line) that maximises both the whole positive and negative predictive values permitted correct classification of all the images, which is in line with previous evidence [44]. It is interesting to note that while allowing identification of the source, the noise left by the sensor has a higher variance in certain cases, resulting in scattered values (Figure 4b,c,i,k,l). This phenomenon is probably due to the different characteristics of the built-in sensors in the different devices and is the reason why, when defining a robust fingerprint, it is necessary to average the residual PRNUs of *n* pictures captured by the device. In the subsequent subsections, we discuss the results of our three experiments (e.g., *profile attribution* and *intra-layer* and *inter-layer user profile linking*) on the images downloaded from the thirteen SNs.

### 7.1. Profile Attribution

The *Profile attribution* task allows for verifying of the smartphone through which a user has taken pictures and uploaded the images to an SN (see case (a) in Figure 1). In this test, for each SN in Table 1 and for each smartphone camera in Table 2 we used a **training set** of 33 original images and a **test set** of 17 downloaded images. This means that we obtained thirteen graphs for each smartphone camera, one for each SN, which appear as as a single graph in Figure 4. In this case, in order to define the threshold that allows classification of the images, we used a simple generalized linear model.

**Figure 5:**
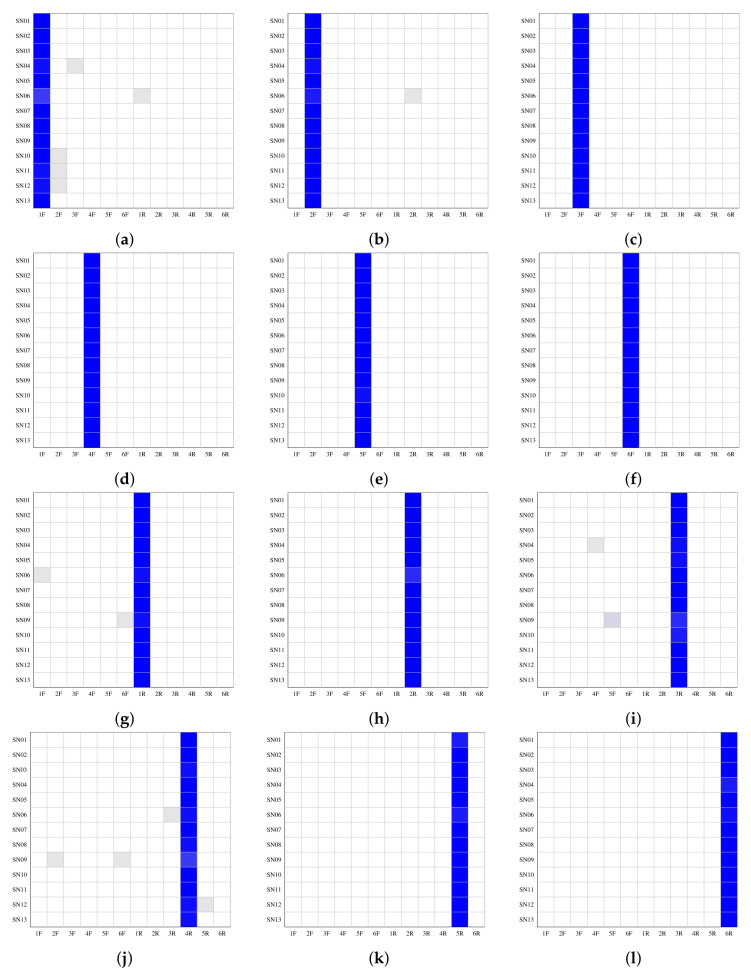
*Profile attribution* results: each graph groups the results of a single source on all thirteen SNs, six for the front cameras (**a**–**f**) and six for the rear cameras (**g**–**l**); the number of images in each cell is identified through a white-to-blue scale, from 0 (white) to 17 (blue).

Figure 5 shows the *profile attribution* results for each smartphone camera; the results for the front cameras are in the first two rows and those for the rear cameras are in the second two rows. In particular, each row in each graph represents the classification results for a specific SN and each white-to-blue scale cell identifies the seventeen images in the test set assigned to the source camera in that SN, from 0 (white) to 17 (blue). This aggregation allows the *user-unaware watermarking* capability of each smartphone camera in each SN to be highlighted. If all of the images are correctly assigned, an intense blue column can be seen in each graph corresponding to the right source and no other “switched on” cells out of that column. The results show that for all source cameras in each SN the fingerprint, FP, permits correct classification of almost all of the seventeen downloaded images. Moreover, the erroneously assigned images do not compromise the identification of the source, as in only in five graphs out of twelve (that is, (a), (b), (g), (i), and (l)) can we see very few images wrongly assigned to the right source.

### 7.2. Intra-Layer User Profiles Linking

In this section, we discuss results in the context of *intra-layer user profile linking*, that is, the task of identifying whether a set of images from two different user profiles within the same SN belong to the same user (see case (b) in Figure 1). For this reason, both the **training set** (33 downloaded images) and the **test set** (17 downloaded images) were taken from the same SN. Thus, we obtained thirteen graphs (one for each SN) for each smartphone camera, and again used a generalized linear model for the classification process.

**Figure 6:**
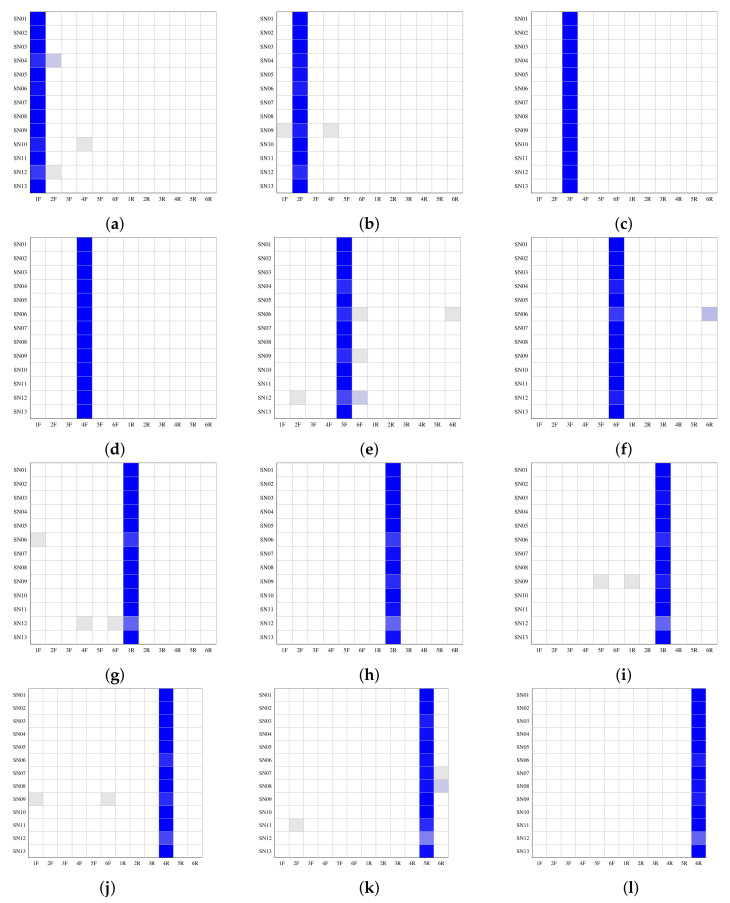
*Intra -layer user profile linking* results: each graph groups the results of a single source on all thirteen SNs, six for the front cameras (**a**–**f**) and six for the rear cameras (**g**–**l**); the number of images in each cell is identified through a white-to-blue scale, from 0 (white) to 17 (blue).

Figure 6 shows the *intra-layer user profile linking* performance of each smartphone camera; front cameras are in the first two rows and rear cameras are in the second two rows. In particular, each row in each graph represents the classification results for a specific SN and each white-to-blue scale cell identifies the images assigned to the source camera in that SN, from 0 (white) to 17 (blue). The results show that for each front source camera the fingerprint, FP, permits correct classification of almost all of the seventeen downloaded images for each SN. WeChat (penultimate row from the top in each graph) returns the worst results for the rear cameras, probably because WeChat utilizes a high compression level for large images. However, in all the graphs a well-defined blue column corresponding to the right source can be seen.

### 7.3. Inter-Layer User Profile Linking

*Inter-layer user profile linking* is the most challenging task, and allows for verification of whether two sets of images from different user profiles on different SNs belong to the same user (see case (c) in Figure 1). As the combination of all SNs produces a very large set of results (i.e., twelve graphs for each SN for each camera), we selected a representative subset of SNs composed of Facebook (SN01), Instagram (SN04), Telegram (SN07), and WhatsApp (SN13). In this case, the **training set** of 33 downloaded images and the **test set** of 17 downloaded images were selected from different SNs among the selected ones. For instance, while we used 33 images from Facebook to define the fingerprint, FP, all of the 17-image test sets were selected from Instagram. Moreover, we used a generalized linear model for the classification process in this test.

**Figure 7:**
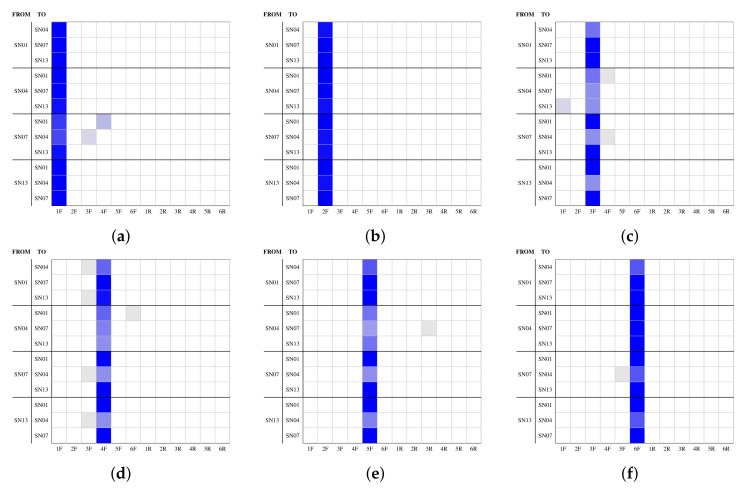

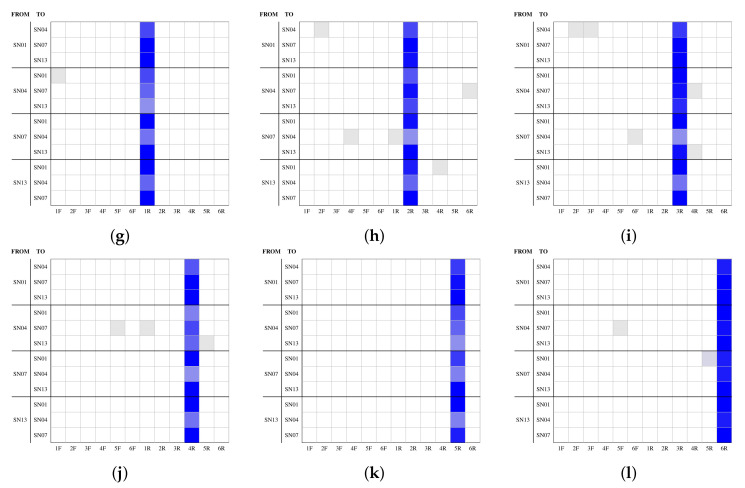
*Inter-layer user profile linking* results: each graph groups the results of a single source on all thirteen SNs, six for the front cameras (**a**–**f**) and six for the rear cameras (**g**–**l**); the number of images in each cell is identified through a white-to-blue scale, from 0 (white) to 17 (blue).

The combination of all the selected SNs produced the results shown in Figure 7; front cameras are in the first two rows and rear cameras are in the second two rows. Each graph represents the *inter-layer user profiles linking* performances for a particular source camera. Specifically, each group of three rows from top to bottom in each graph represents the results using the fingerprint from a specific SN (i.e., Facebook for the first three rows, Instagram for the second three rows, Telegram for the third three rows, and WhatsApp for the last three rows). The results are slightly worse in comparison to *intra-layer user profile linking*; however, the number of misclassified images is very low. The problem is particularly significant when Instagram images are involved in the comparison. In fact, the worst results occurred when the fingerprint was defined using Instagram images (i.e., the second group of three rows from the top) or when attempting to classify Instagram images (i.e., the first row in the first group of three rows and the second row in the third and fourth group of three rows). The slightly worse results obtained with Instagram are probably due to the higher compression rate applied by the platform (i.e., up to 94.14% for the images from the smartphones used in these experiments). A possible method for improving these results involves using a larger number of images to define the fingerprint of the device. However, all the other SN combinations that do not involve Instagram images obtained good results, especially for the front camera images.

These results show the effectiveness of the PRNU-based method for the task of defining a *user-unaware watermark* for all considered SNs. It is sufficiently robust in spite of using shared images degraded by the SNs during the uploading and downloading process. Our results indicate that the proposed method can be successfully used for both *profile attribution* and for *intra/inter-layer user profile linking*. Moreover, the method makes it possible to identify those profiles that belong to the same user which can, for instance, permit the identification of fake profiles.

## 8. Discussion

The authorship attribution and verification of images shared online is becoming more and more important, raising questions ranging from privacy and security issues to forensic implications. Here, we explored different techniques for proving ownership of images shared on SNs through image watermarking techniques. In particular, we investigated whether SNs mark the images being uploaded on them (i.e., *social network watermarking*); we analysed conventional watermarking algorithms as a tool for reliable marking of images to be uploaded on SNs (i.e., *user-explicit watermarking*); finally, we explored iwhether the PRNU of the camera through which the image was taken can be used to create a watermark which is robust enough to be preserved on SNs (i.e., *user-unaware watermarking*).

The *social network watermarking* test revealed that none of the thirteen SNs we investigated performs watermarking techniques on images or applies any profile-dependent watermark visible outside the network. The *user-explicit watermark* test underlined that none of the thirteen conventional watermarking techniques considered can be successfully used across all SNs or generate a result unnoticeable by the user. Finally, the *user-unaware watermark* test revealed the effectiveness of PRNU-based methods for the task of creating a sort of implicit watermark that is robust enough to address different crucial subtasks, such as *profile attribution* and *intra* and *inter-layer user profile linking*.

The smartphone fingerprint defined via the PRNU-based method is well-known in the literature, and the proposed solution has shown promising results in the context of authorship attribution and verification of images shared on SNs. Although classification errors remain, both the potential and applicability of the proposed method are evident. In particular, we believe that our results can be further improved by approximating the device fingerprint using more images, especially in light of the large number of images shared on SNs every day. This can represent a solution for the issues encountered with Instagram results involving the relatively more penalising impact of compression on that SN.

It is worth noting that the proposed method can be extended to other devices equipped with a camera, such as digital cameras [86] and webcams [87]. In particular, the results obtained here hold promise for integration within a multi-factor user authentication framework that could enhance user security and produce the digital equivalent of validation via biometric features [88], where the authentication scheme is based on the verification of the possession of one’s own smartphone. Moreover, the notion of “digital-metric” features for authentication can be further strengthened using other fingerprints extracted from the other built-in sensors of the device, such as the microphone [89] and accelerometer [6], among others.

## 9. Conclusions

An increasingly large amount of data is uploaded daily for sharing on SNs; as the majority of this flow of data includes images, it is thus important to find solutions for authorship attribution and verification of these pictures to avoid problems such as impersonation and fake profiles.

We performed multiple investigations in the domain of image watermarking. All of the experiments we conducted in the context of *social network watermarking* showed no evidence of watermarks. While Facebook introduces certain suspicious values to image metadata, this cannot be considered for a robust watermark in the SN context.

Next, we conducted a detailed review of how conventional image watermarking algorithms (i.e., *user-explicit watermarking*) behave on each selected SN. In particular, we examined whether they can resist the compression algorithms applied by social platforms. We found that none of the algorithms were able to apply a robust watermark retained in all thirteen of the selected SNs. Moreover, in certain cases these watermarking algorithms visually altered the original image.

Finally, we showed that a *user-unaware watermark* based on PRNU is able to resist the compression algorithms of all thirteen SNs investigated in our study. The PRNU is a regular and systematic noise component characterizing each image captured by off-the-shelf cameras, including the smartphone’s camera. We proved that the method based on this user-unaware trace allows several challenging tasks to be performed, namely, *profile attribution* and *intra* and *inter-layer user profile linking*. Moreover, we showed how a *user-unaware watermark* can be exploited for *fake profile detection* as a corollary of the three previous tasks.

## Figures and Tables

**Table 1:** Social Networks used for investigation activities and the default pixel resolution accepted by each platform.

ID	Social Networks	Image Sizes
SN01	Facebook	2048 × 1152
SN02	Flickr	2048 × 1152
SN03	Google Currents	2048 × 1152
SN04	Instagram	1080 × 1080
SN05	LinkedIn	2048 × 1152
SN06	Pinterest	2048 × 1152
SN07	Telegram	1280 × 720
SN08	Tumblr	1280 × 720
SN09	Twitter	2048 × 1152
SN10	Viber	1280 × 720
SN11	VK	2560 × 1440
SN12	WeChat	1280 × 720
SN13	WhatsApp	1600 × 1200

**Table 2:** The six smartphones used for the user-unaware watermarking tests and the pixel resolution of the front and rear cameras.

ID	Brand	Model	Front Camera	Rear Camera
1	Apple	iPhone 6	1280 × 960	3264 × 2448
2	Apple	iPhone 6 Plus	1280 × 960	3264 × 2448
3	LG	Nexus 5	1280 × 960	3264 × 2448
4	LG	Nexus 5	1280 × 960	3264 × 2448
5	Samsung	Galaxy S2	1600 × 1200	3264 × 2448
6	Samsung	Galaxy S2	1600 × 1200	3264 × 2448

**Table 3:** The results of watermark extraction. For each algorithm, the failure/success of the extraction process is shown with a triplet of ∘/• symbols following a sequence standard (see Column III in Table 1) of large (4128 × 2322) and small (640 × 480) resolution images.

SNs	A1 [77]	A2 [77]	A3 [77]	A4 [77]	A5 [77]	A6 [77]	A7 [78]	A8 [79]	A9 [80]	A10 [81]	A11 [82]	A12 [83]	A13 [84]
Facebook	∘,∘,∘	∘,∘,∘	∘,∘,∘	∘,∘,∘	∘,∘,∘	∘,∘,∘	∘,∘,∘	∘,∘,∘	∘,∘,∘	•,•,•	•,∘,∘	∘,∘,∘	∘,∘,∘
Flickr	•,•,•	•,•,•	•,•,•	•,•,•	•,•,•	•,•,•	•,•,•	•,•,•	•,•,•	•,•,•	•,∘,•	•,•,∘	•,∘,•
Google Currents	•,•,•	•,•,•	•,•,•	•,•,•	•,•,•	•,•,•	•,•,•	•,•,•	•,•,•	•,•,•	•,∘,•	•,•,∘	•,∘,•
Instagram	∘,∘,∘	∘,∘,∘	∘,∘,∘	∘,∘,∘	∘,∘,∘	∘,∘,∘	∘,∘,∘	∘,∘,∘	∘,∘,∘	∘,∘,∘	∘,∘,∘	∘,∘,∘	∘,∘,∘
LinkedIn	∘,∘,∘	∘,∘,∘	∘,∘,∘	∘,∘,∘	∘,∘,∘	∘,∘,∘	∘,∘,∘	∘,∘,∘	∘,∘,∘	∘,∘,∘	∘,∘,∘	∘,∘,∘	∘,∘,∘
Pinterest	∘,∘,∘	∘,∘,∘	∘,∘,∘	∘,∘,∘	∘,∘,∘	∘,∘,∘	∘,∘,∘	∘,∘,∘	∘,∘,∘	∘,∘,∘	•,∘,•	∘,∘,∘	∘,∘,∘
Telegram	∘,∘,∘	∘,∘,∘	∘,∘,∘	∘,∘,∘	∘,∘,∘	∘,∘,∘	•,∘,•	∘,∘,∘	∘,∘,∘	∘,∘,∘	•,∘,•	∘,∘,∘	∘,∘,∘
Tumblr	∘,∘,•	∘,∘,•	∘,∘,•	∘,∘,•	∘,∘,•	∘,∘,•	•,∘,•	•,•,•	•,•,•	•,•,•	•,∘,•	∘,∘,∘	∘,∘,•
Twitter	∘,∘,∘	∘,∘,∘	∘,∘,∘	∘,∘,∘	∘,∘,∘	∘,∘,∘	∘,∘,∘	∘,∘,∘	∘,∘,∘	∘,∘,∘	•,∘,•	∘,∘,∘	∘,∘,∘
Viber	∘,∘,∘	∘,∘,∘	∘,∘,∘	∘,∘,∘	∘,∘,∘	∘,∘,∘	∘,∘,•	•,•,•	∘,∘,∘	•,•,•	∘,∘,•	∘,∘,∘	∘,∘,∘
VK	∘,∘,∘	∘,∘,∘	∘,∘,∘	∘,∘,∘	∘,∘,∘	∘,∘,∘	•,∘,•	•,∘,•	∘,∘,∘	∘,∘,∘	•,∘,•	∘,∘,∘	•,∘,∘
WeChat	∘,∘,∘	∘,∘,∘	∘,∘,∘	∘,∘,∘	∘,∘,∘	∘,∘,∘	∘,∘,•	•,•,•	∘,∘,∘	•,•,•	∘,∘,•	∘,∘,∘	∘,∘,∘
WhatsApp	∘,∘,∘	∘,∘,∘	∘,∘,∘	∘,∘,∘	∘,∘,∘	∘,∘,∘	•,∘,•	∘,∘,∘	∘,∘,∘	•,•,•	•,∘,•	∘,∘,∘	•,∘,•

## Data Availability

The dataset is available from: http://smartdata.cs.unibo.it/datasets#images, accessed on 9 May 2022.
